# Long-Term Dynamic Changes in Cosmetic Outcomes and Patient Satisfaction after Implant-Based Postmastectomy Breast Reconstruction and Contralateral Mastopexy with or without an Ultrapro Mesh Sling Used for the Inner Bra Technique. A Retrospective Correlational Study

**DOI:** 10.3390/cancers13010073

**Published:** 2020-12-29

**Authors:** Zsófia József, Mihály Újhelyi, Orsolya Ping, Szilárd Domján, Rita Fülöp, Gabriella Ivády, Ráhel Tislér, Gábor Rubovszky, Norbert Mészáros, István Kenessey, Zoltán Mátrai

**Affiliations:** 1 St. Imre Teaching Hospital, Department of Plastic Surgery, 12-16. Tétényi Rd., 1115 Budapest, Hungary; zsofia.jozsef@gmail.com; 2 National Institute of Oncology, Department of Breast and Sarcoma Surgery, 7-9. Ráth György Str., 1122 Budapest, Hungary; ping.orsolya@oncol.hu (O.P.); domjan.szilard@oncol.hu (S.D.); matraidok@oncol.hu (Z.M.); 3 National Institute of Oncology, Centre of Radiological Diagnostics, 7-9. Ráth György Str., 1122 Budapest, Hungary; fuloprita@outlook.com; 4 National Institute of Oncology, Centre of Pathology, 7-9. Ráth György Str., 1122 Budapest, Hungary; ivadygabi@oncol.hu; 5 Faculty of Medicine, Semmelweis University, 7-9. Ráth György Str., 1122 Budapest, Hungary; rahelannatisler@gmail.com; 6 National Institute of Oncology, Department of Oncological Internal Medicine and Clinical Pharmacology, 7-9. Ráth György Str., 1122 Budapest, Hungary; garub@oncol.hu; 7 National Institute of Oncology, Centre of Radiotherapy, 7-9. Ráth György Str., 1122 Budapest, Hungary; meszarosnorbert@oncol.hu; 8 National Institute of Oncology, National Cancer Registry, 7-9. Ráth György Str., 1122 Budapest, Hungary; kenessey.istvan@oncol.hu; 9 Second Department of Pathology, Semmelweis University, 26. Üllői Str., 1085 Budapest, Hungary

**Keywords:** breast reconstruction, mastopexy, oncoplastic breast surgery, breast symmetrization

## Abstract

**Simple Summary:**

Immediate implant-based postmastectomy breast reconstruction (IPMBR) with contralateral symmetrization has mostly short-term limited evidence of cosmetic outcomes. Ageing after IPMBR and symmetrization may contribute to symmetry worsening. This non-interventional retrospective correlational study presents the clinical and aesthetic results of synthetic ULTRAPRO^®^ mesh inner bra sling symmetrization mastopexy with standard mastopexies. A total of 59 patients were enrolled in the mesh group (MG), and 58 patients were enrolled in the non-mesh group (NMG). There were no significant differences in surgical complications (*p* = 0.521; chi-square. The median sternal notch-to-nipple distance difference was 1 cm in the MG and 3.5 cm in the NMG from the last follow-up, and the median nipple-to-inferior mammary fold distance differences were 0.5 cm and 0.75 cm. ULTRAPRO^®^ mesh sling symmetrization can be successfully used to decrease pseudoptosis and nipple down-migration, offering a safe alternative for long-lasting symmetry and high patient satisfaction.

**Abstract:**

Immediate implant-based postmastectomy breast reconstruction (IPMBR) with contralateral symmetrization has mostly short-term limited evidence of cosmetic outcomes. Because 84% of early-stage breast cancer patients have overall survival of more than 10 years, reconstructed breast symmetry should provide long-lasting results and acceptable patient satisfaction. Ageing, changes in body weight, and biomechanical changes after IPMBR and symmetrization may contribute to symmetry worsening. This non-interventional single-centre retrospective correlational study presents the clinical and aesthetic results of synthetic ULTRAPRO^®^ mesh inner bra sling symmetrization mastopexy with standard mastopexies. According to the results, a total of 59 patients were enrolled in the mesh group (MG), and 58 patients were enrolled in the non-mesh group (NMG). There were no significant differences in surgical complications (*p* = 0.521; chi-square). The median sternal notch-to-nipple distance difference was 1 cm in the MG and 3.5 cm in the NMG from the last follow-up, and the median nipple-to-inferior mammary fold distance differences were 0.5 cm and 0.75 cm. The mesh did not hinder the follow-up investigation. In conclusion, ULTRAPRO^®^ mesh sling symmetrization can be successfully used to decrease pseudoptosis and nipple down-migration, offering a safe alternative for long-lasting symmetry and high patient satisfaction.

## 1. Introduction

The goal of modern breast reconstruction surgery is to immediately replace the missing breast volume, reshape the distorted contours, and create optimal symmetry by modelling the contralateral breast. Reconstructive surgeries including contralateral symmetrization techniques are well-known rehabilitation procedures associated with significant psycho-oncological benefits, high patient satisfaction, and low complication rates [1,2]. Currently, aesthetic and symmetrical breasts are realistic expectations for most breast cancer patients following all kinds of postmastectomy breast reconstruction (PMBR). Nonetheless, the time value of the cosmetic outcome seems to be more problematic with limited clinical and patient-related outcomes in the long term [3]. In fact, symmetrization techniques have only mid-level and short-term evidence according to oncological and cosmetic outcomes. Henceforth, maintaining long-lasting symmetry remains a challenge, basically because of mainstream immediate implant-based postmastectomy breast reconstruction (IPMBR) on the cancerous side versus the different structure of the natural breast on the contralateral side [4,5].

Symmetrization surgeries are commonly performed as a second operation after IPMBR and adequate adjuvant therapies [6]. Currently, complete IPMBR can be performed at the time of mastectomy for one-stage reconstruction with contralateral symmetrization using pre- or subpectorally placed implants in combination with biological or synthetic meshes [7,8]. Symmetrization techniques include mastopexy, breast reduction, silicone implant augmentation with or without mastopexy, autologous fat grafting, and implant-based reconstruction combined with advanced prophylactic mastectomies (skin-sparing mastectomy, SSM; areola-sparing mastectomy, ASM; nipple-sparing mastectomy, NSM; skin-reducing nipple-sparing mastectomy, SRNSM) corresponding to the tumor side and patient-related factors [9]. It is well known that the most obvious symmetry can be achieved by the same surgical technique performed on both sides (e.g., bilateral NSM and implant-based reconstruction or therapeutic mammoplasty with contralateral inverted-T reduction mastopexy) [10].

**Figure 1:**
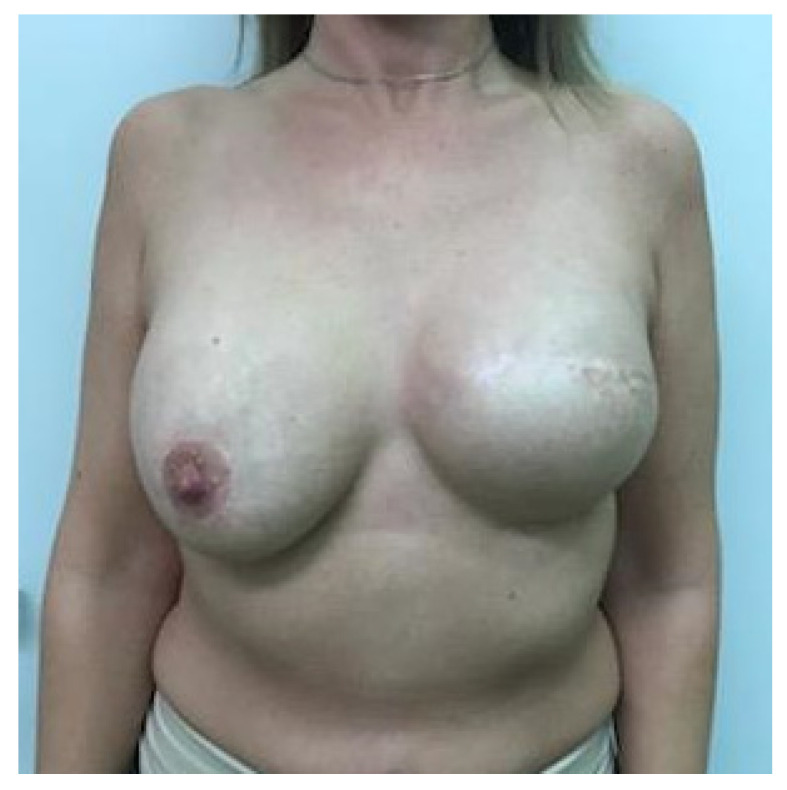
Asymmetry after one year of implant-based IPMBR on the left side and mastopexy and implant symmetrization surgery.

It has been shown that 84% of early-stage breast cancer patients can achieve disease-free survival of 10 years with a combination of multidisciplinary treatments [11]. Therefore, breast symmetrization surgery should provide long-lasting results, especially if one-stage IBR and contralateral symmetrization are performed. Ageing, changes in body weight (e.g., weight gain in the context of up to 10 years of endocrine therapy), stretching of tissues, biomechanical changes after implantation, postirradiation fibrosis, and/or different degrees of breast lymphedema may necessitate further and sometimes repeated reconstructive and symmetrization procedures (re-mastopexy, autologous fat grafting on the mastectomy or symmetrization side, minor corrections, capsuloplasty), in combination with additional physical and emotional burdens for patients as well as costs and caseloads for health systems. Another possible reason for the significant decrease in breast symmetry is the fact that over time, the natural breast develops differently than the reconstructed breast. The shape of the reconstructed breast remains constant 3–6 months after IPMBR because of the fixed volume and shape of the silicone implant, which dominates the breast with a thin overlying soft tissue layer. Due to the clearly different biological properties (e.g., gravity causes ptosis of the uplifted breast after mastopexy, upper pole flattening, lower pole expansion (pseudoptosis), hormone therapy may increase the volume of the natural breast, while the implant-based reconstructed breast does not show the same change, progressive capsular contracture on the mastectomy side, nipple flattening, nipple tattoo fading), the two breasts become increasingly different with time, resulting in asymmetry and decreased patient satisfaction, see Figure 1.

On the symmetrization side, pseudoptosis or the increase in the sternal notch-to-nipple distance becomes visible within months [12]. Revision surgery aims to change the position of the nipple-areola complex (NAC) by removing excess skin, reshaping the parenchyma, reducing the horizontal and vertical planes, and enhancing the projection of the breast [13].

To prevent pseudoptosis or elongation of the sternal notch-to-nipple distance, the parenchyma should be fixed to the chest wall by using a so-called internal bra, dermal bra, dermal strips technique, superficial fascial suspension, fascia lata, or muscular slings technique [12,14,15,16]. These slings are able to hold the weight of the remnant breast parenchyma and thus relieve the lower pole skin of the breast from continuous pressure. Biological materials such as acellular dermal matrices (ADMs) and non-biological materials can be used for internal bra sling techniques. Non-biological materials have been recently introduced for IBR as low-cost alternatives to ADMs and include the polyglactin 910 (Vicryl^®^) mesh TiLOOP^®^ Bra, SERAGYN^®^ BR mesh, and the TIGR^®^ Matrix [17,18,19,20].

This study presents an evaluation of synthetic ULTRAPRO^®^ mesh in mastopexy procedures. The ULTRAPRO^®^ mesh is a partially absorbable, lightweight synthetic material manufactured from approximately equal parts of absorbable poliglecaprone-25 monofilament fibres and non-absorbable polypropylene monofilament fibres. It is extensively used in surgery, especially in hernia repair. The Monocryl part (poliglecaprone) absorbs within 90 to 120 days, whereas the lightweight polypropylene mesh has a pore size of 3 to 4 mm [21].

According to the hypothesis of the study, the results of implant-based IPMBR and symmetrization following advanced postmastectomy techniques significantly decrease over time and later result in a limited patient satisfaction rate. The aim is to present the ULTRAPRO^®^ mesh sling technique combined with mastopexy and use correlation analysis to dynamically measure objective changes, quality of life, and patient satisfaction associated with the symmetry achieved by different surgical techniques at the end of the follow-up.

## 2. Patients and Methods

This was a non-interventional single-centre clinical retrospective study and was performed with the permission of the Local Ethical Committee of the National Institute of Oncology. The approval number is EtikaBiz. Ujhelyi Mihaly 101/1 September 2020, approval date: 1 September 2020. The inclusion period for this retrospective study was between 1 January 2017, and 31 May 2020.

In this study, the standardized surgical techniques used are considered to be routine procedures in the literature and in the praxis of the authors. The surgical techniques were performed by four specialized oncoplastic breast surgeons. The applied oncological therapies were unaffected by the study and followed up-to-date institutional treatment protocols according to the European Society of Medical Oncology (ESMO) guidelines [22].

Patients with unilateral primary breast cancer (clinical stage 0–III); without a history of any breast surgeries on both sides; who were indicated for advanced SSM, NSM, or ASM independently of the axillary surgery; and who had immediate or delayed-immediate implant-based reconstruction on the ipsilateral side and symmetrization on the contralateral side (mastopexy/reduction and/or implant-based reconstruction and/or breast sling with ULTRAPRO mesh) were selected.

Selection of the surgical technique was based on decision-making models according to institutional practice, and selection was made individually based on the patient’s oncological disease (e.g., nipple removal) and patient-related factors, such as breast size, skin quality, comorbidities (diabetes), smoking, and ptosis degree (NSM vs. SSM). The method of routine breast surgery is not different for patients within or outside the study. The principles for selecting symmetrization techniques were based on the individual decision of the breast surgeon. In addition, reinforcement with the ULTRAPRO^®^ mesh was used during mastopexy when the degree of breast ptosis was at least grade 3 or with cup size C according to the Regnault classification or when the patient had significant pseudoptosis, so the fibrous ligament system and the skin envelope of the breast had a very loose structure [23].

Expander replacement with a silicone implant and contralateral breast symmetrization were performed at least six months after the completion of adjuvant radiotherapy and at least three months after adjuvant chemotherapy. Before the surgeries, body measurements and photo documentation were always precisely performed as routine oncoplastic follow-up examinations. To compare the postoperative aesthetic results of the sling, patient data were only used when nipple reconstruction and NAC tattooing were performed. The follow-up was performed at the time of regular breast surgical follow-up examinations, with three monthly for two postoperative years and six monthly thereafter until the fifth postoperative year.

In the prospectively led institutional database, the recorded data were age, body mass, height, BMI, pre-/postoperative cup sizes, presence of preoperative breast asymmetry and its severity, past history, medication, smoking habits, oncological data, cTNM, pTN, pathological data, molecular genetic subtype, neoadjuvant and adjuvant therapy, and type of axillary treatment. The following plastic surgical parameters were documented from the first to the third week and at the end of the follow-up: the sternal notch-to-nipple, nipple-to-midline, and nipple-to-inferior mammary fold (IMF) distances; the vertical and horizontal widths of the areola; and the Regnault classification of breast ptosis.

Postoperative complications were classified following the Clavien–Dindo classification [24,25]. Grade I complications (light inflammation, non-surgical haematoma or suffusion, seroma formation, partial skin/NAC loss, limited fat necrosis, SSI, and lymphedema) do not require medication or surgical treatment. Grade II complications are grade I complications that require medication or surgical interaction (antibiotic therapy, resuturing due to SSI and multiple punctures due to chronic seroma). Grade III complications require invasive surgical action (haematoma evacuation, chronic inflammation that requires reoperation, severe fat necrosis, full skin/NAC necrosis, and wound dehiscence). Grade IV complications indicate temporary organ failure. Grade V complications are those that lead to death.

The postoperative data of the BREAST-Q questionnaire and 5-point Likert scale were noted only at the end of the follow-up [26]. Quality of life was measured by a validated BREAST-Q questionnaire. For this questionnaire, a score on a 1–100 scale was given, measuring the variables of “satisfaction with the breast”, “psychosocial wellbeing”, “physical wellbeing: chest”, and “sexual wellbeing”. Higher rates indicate better quality of life. [27]

Likert scales were used (1: definitely not, 2: no, 3: abstain, 4: agree, 5: definitely agree) to evaluate the subjective aesthetic outcome based on the photo documentation (preoperative and after delayed reconstruction with symmetrization) at the end of the follow-up. Based on the photo documentation, three non-involved breast surgeons performed the evaluations separately without communication. The results were collected and averaged.

Control mammography, breast ultrasound, and breast magnetic resonance imaging (MRI) results and imaging were retrospectively examined by breast radiology specialists.

Categorical data were compared using the chi-square or Fisher’s exact test. Asymmetrical numeric data were analyzed with the Mann–Whitney test or Wilcoxon signed rank test for matched samples. The Wald–Wolfowitz runs test was applied in the analysis of the questionnaire to minimize type I error. Statistical significance was confirmed when *p*-values were <0.05. Statistical analysis was performed using Statistica 13.5 software (TIBCO Software Inc, Palo Alto, CA, USA).

### Surgical Therapy for Inferior or Modified Central Pedicled Inverted-T Mastopexy with ULTRAPRO^®^ Sling Mesh

In this study, the standardized inferior pedicle or the modified central pedicle Wise mastopexy technique from an inverted-T skin incision was performed [28]. An extra ULTRAPRO^®^ mesh sling was placed over the parenchyma and covered with fascio-cutaneous flaps to suspend the breast if ptosis was suspected. The surgical technique was first introduced and reported by Zoltán Mátrai.

The inferior pedicle, inverted-T breast reduction technique was first described by Ribeiro in 1975 [29]. The most important advantages of this technique are the safe preservation of the NAC circulation by the inferior pedicle, especially in remarkably large-sized breasts, in addition to filling the volume of the upper pole of the breast by the parenchyma of the lower hemisphere. Planning and implementation of the procedure require plastic surgical or oncoplastic knowledge.

**Figure 2:**
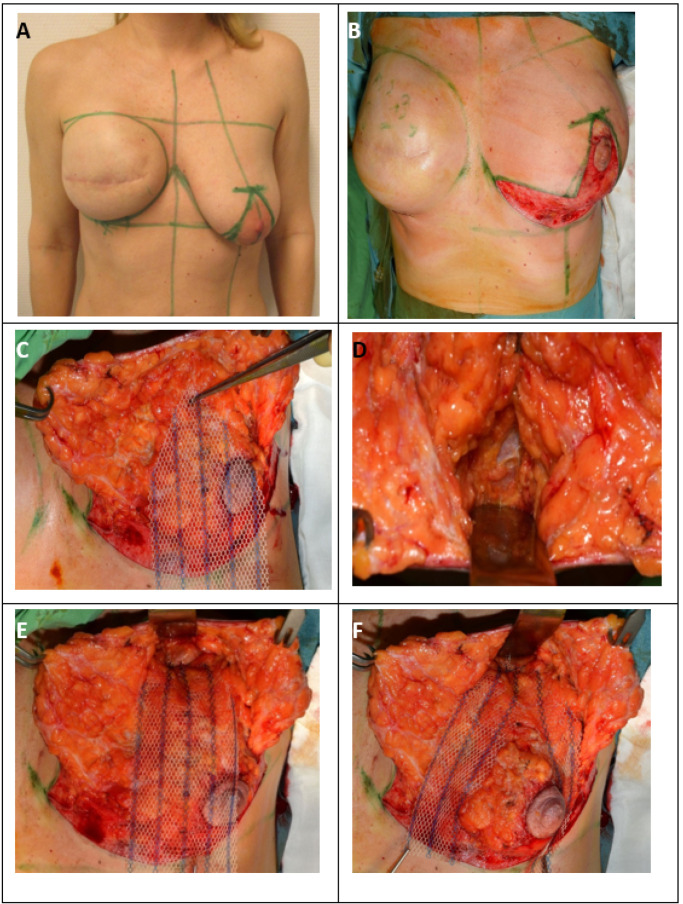

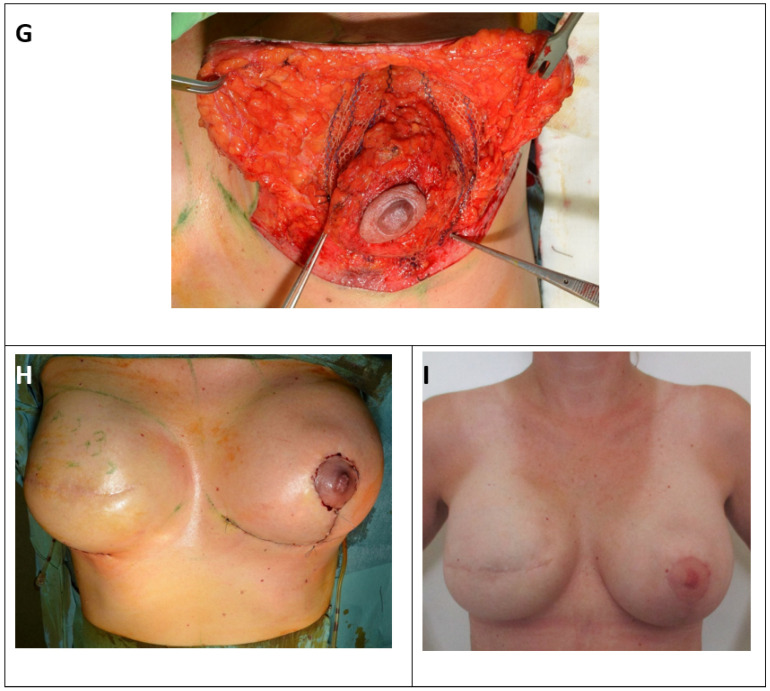
Surgical therapy for inferior or modified central pedicled inverted-T mastopexy with ULTRAPRO^®^ sling mesh (**A**) Preoperative drawings of inverted-T symmetrization mastopexy on the left side and expander-to-implant changes on the right side. (**B**) Skin reduction via de-epithelization of the preoperatively marked skin on the lower pole of the left breast. (**C**) Medial and lateral fascio-cutaneous flaps are elevated from the breast parenchyma, and the Ultrapro mesh is placed towards the upper pole of the breast. (**D**) The fascia of the pectoralis major muscle at the upper pole is prepared as the point of mesh fixation. (**E**) ULTRAPRO mesh sling adapted with double absorbable sutures to the pectoralis major fascia of the upper pole. (**F**) The mesh sling was modelled in “trouser-form” and placed in omega-form around the de-epithelialized parenchyma for suspension. (**G**) The divided mesh slings are adapted via absorbable sutures on the lower pole and medially and laterally on the sides of the parenchyma for flexible fixation. (**H**) Cosmetic outcome after skin closure. (**I**) 15-month postoperative result.

Preoperative markings were made in the standing position (Figure 2a).

A single dose of antibiotic (cefazolin or clindamycin) was administered 30–60 min before surgery. After prepping and draping, along the incision lines, the subdermal plexus was infiltrated with a 0.5% solution of epinephrine and lidocaine.

Next, the NAC was outlined with an appropriately sized areola marker, and the epidermis was transected along the incision line (except for the skin of the neo-areola). Before cutting through the full thickness of the skin, it was best to de-epithelialize at least a 5–10-mm-wide zone on every edge of skin to avoid necrosis of the marginal line. The next step was de-epithelization of the preoperatively marked skin area on the lower breast pole (Figure 2b).

The de-epithelized dermis was transected with an electrosurgical device 5–10 mm from the markings along the vertical line, and the transection was continued on the lower horizontal and distal wound edges in the IMF. In the case of a modified central pedicled wide-pattern technique, it was necessary to leave the dermis surrounding the NAC intact (with at least a 10-mm margin), facilitating its blood supply from the subdermal plexus, which mainly originates from the perforator vessels running in the horizontal Würingers fascia [28]. On the vertical wound edges elevated by skin hooks, dissection was performed over the layer of superficial fascia in the lamina anterior towards the superficial fascia of the pectoralis major muscle in both the medial and lateral directions (Figure 2c). Considering the size of the contralateral breast, adequate reduction of the upper parenchyma hemispherium was performed. The residual breast tissue and NAC were supplied by the perforating vessels of Würinger’s septum. The vascular supply of the medial and lateral dermal flaps was derived from the perforators located superiorly in the second/third intercostal space and medially from the parasternal perforators located in the second and/or third intercostal spaces, as well as from the supplying vessels from the lateral axillary region. The defect caused by the reduction was easily closed with surrounding parenchyma pillars using simple interrupted 2.0 absorbable sutures.

In the case where ptosis was suspected, an additional non-biological ULTRAPRO^®^ mesh sling was used in the subcutaneous layer to suspend the breast (Figure 2c). 

The size of the ULTRAPRO^®^ mesh that was used was 75 × 150 mm. The mesh was tailored to a trouser form. The upper part of the trouser-tailored mesh was adapted with double absorbable sutures to the pectoral muscle according to the upper pole at the 12 o’clock position (approximately the second rib) (Figure 2d,e).

The trouser legs placed in the omega form medially and laterally encircled the breast parenchyma, and the lower parts were fixed to the lower pole of the breast tissue (Figure 2f). 

The middle parts of the legs were fixed medially and laterally to the breast tissue (Figure 2g). 

Attention should be paid when performing lower fixation. The end of the mesh should be placed approximately 2 cm higher than the inverted-T sutures to avoid fistulas if necrosis of the skin edges occurs. Prior to wound closure, a 16Ch suction drain was placed from a separate orifice. Next, approximation and closure of the dermis and subcutaneous tissue with simple interrupted absorbable sutures were performed. Monofilament continuous 4.0 non-absorbable sutures were used for skin closure of the periareolar wound, and continuous 3.0 non-absorbable sutures were used for the horizontal and vertical incisions in both layers (Figure 2h). 

Finally, the wounds were coated with Bactroban^®^ gel and covered with a semipermeable Mepore^®^ dressing (Figure 2i).

## 3. Results

Five patients were lost to follow-up. As a result, a total of 59 patients were enrolled in the mesh group (MG), and 58 patients were enrolled in the non-mesh group (NMG). The median follow-up time was 16 months (range: 1–40 months) in the MG and 19 months (range: 1–38 months) in the NMG. The patient and tumor characteristics are listed in Table 1. 

There were no significant differences in the patient or tumor characteristics. Because of the low number of diabetes mellitus cases the comorbidities as hypertonia, diabetes mellitus, and hypothyreosis were pooled. There were no significant differences is comorbidities between two groups (*p* = 0.221; chi-square). At the last follow-up, there were no cases of local recurrence, while two distant metastases were revealed (one liver, one bone metastasis), one in each group.

The surgical procedure-related characteristics are summarised in Table 2. 

There were no significant differences in the duration of surgery (*p* = 0.371; Mann–Whitney), size of the implants used on the symmetrization side (*p* = 0.055; Mann–Whitney), complications of surgery (*p* = 0.521; chi-square), preoperative breast cup size (*p* = 0,083; chi-square), or postoperative breast cup size (*p* = 0.33; chi-square). There were no cases of mesh rejection, stiffness according to the mesh position, fistula, or chronic seroma formation observed. There was no significant correlation between the comorbidities and complication on mastectomy or symmetrization side (*p* = 0.477 and *p* = 0.239; chi-square). There was no significant correlation between the BMI and complication on mastectomy or symmetrization side (*p* = 0.904 and *p* = 0.535; chi-square). There was no significant correlation between the smoking habits and complication on mastectomy or symmetrization side (*p* = 0.721 and *p* = 0.672; chi-square). Significantly more reduction mastopexy procedures were performed in the MG than in the NMG (*p* = 0.032; chi-square), and significantly larger implants were used on the mastectomy side in the MG (*p* = 0.012; Mann–Whitney).

According to the BREAST-Q questionnaire, significant differences were observed in breast satisfaction, physical wellbeing, and sexual wellbeing. Higher scores were found in patients in the MG, see Table 3.

According to the Likert scale, the average score for the MG was 4.4, and for the NMG was 3.8.

The pre- and postoperative sternal notch-to-nipple, nipple-to-midline, and nipple-to-IMF distances are listed in Table 4.

**Figure 3:**
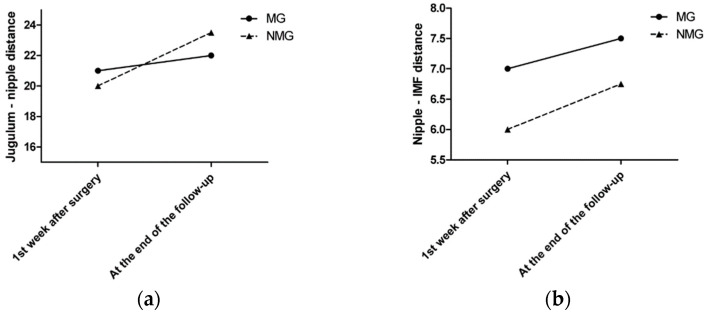
(**a**) The median sternal notch-to-nipple distance difference by time. (**b**) The median nipple-to-IMF distance difference by time.

The median sternal notch-to-nipple distance difference from the symmetrization surgery to the last follow-up was 1 cm in the MG and 3.5 cm in the NMG. The median nipple-to-IMF distance difference from the symmetrization surgery to the last follow-up was 0.5 cm in the MG and 0.75 cm in the NMG (Figure 3a,b).

**Figure 4:**
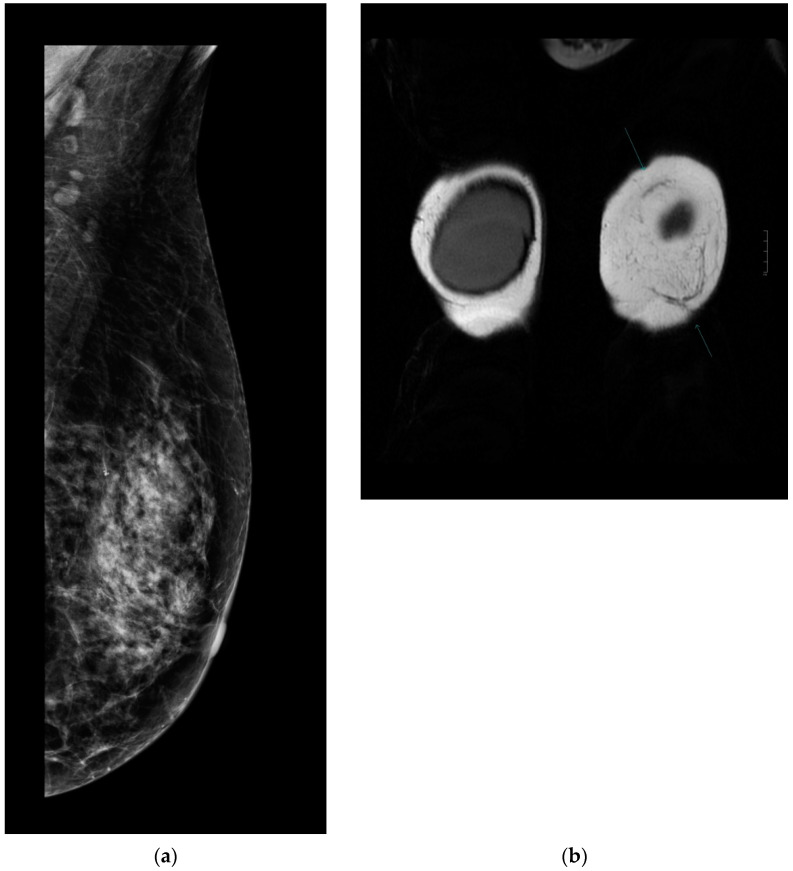
(**a**)**.** Follow-up mammography after ULTRAPRO sling mastopexy. The mesh is not visible on the mammography images; therefore, it does not influence the oncological surveillance. (**b**)**.** Arrows show the mesh on contrast-enhanced MRI. The mesh appears clearly/well depicted on the MR (coronal T2 weighted) images without disturbing the evaluation of the parenchyma.

Every patient underwent control complex breast imaging (mammography as well as breast and axillary US) according to the ESMO guidelines for treatment and follow-up. Breast MRI was performed in 32 patients: 17 in the MG and 15 in the NMG. According to the control breast imaging examinations, the mesh did not disturb the clear visualization or oncological control of the underlying parenchyma (Figure 4a,b).

After 12 months, there were no signs of chronic seroma formation or any other visualization disturbing the lesion on the symmetrization side if the mesh was used.

## 4. Discussion

One of the most reported breast surgical topics is to analyze the optimal techniques of modern mastectomies and IPMBRs using pre- or subpectorally placed implants in combination with biological or synthetic meshes [7,8]. There is plenty of mid-level evidence that these techniques are able to provide safe oncological and high cosmetic outcomes [30,31]. One of the main advantages of modern breast reconstructive techniques is the ability to achieve immediate high-quality complete breast restoration in order to prevent the transitional period without a breast, to prevent the use of a partially filled tissue expander and to alleviate expander-to-implant changes that cause a second operation. However, studies analyzing the durability of breast symmetry and patient-related outcomes, as well as the factors of ageing, weight gain, and tissue elasticity changes that result in asymmetry and consequential patient dissatisfaction, are rare. The hypothesis is that the oncological results of early-stage breast cancers are superior to the relatively short-term results of implant-based PMBRs. This study aims to provide quality data about a surgical technique using a subcutaneous inner bra to prevent breast ptosis as well as consequential asymmetry and patient dissatisfaction.

To strengthen the structure of the lower pole of the breast and decrease nipple displacement, polypropylene meshes or even ADMs for breast symmetrization can be used. The partial absorbable ULTRAPRO^®^ mesh sling as an internal bra seems to provide long-lasting suspension of the breast tissue without significant morbidities such as mesh spool, hardening, extrusion, or prevention of imaging follow-up. According to a review of the indexed international literature, there are no studies that numerically quantify the postoperative sternal notch-to-nipple, nipple-to-midline, or nipple-to-IMF distance after mastopexy, with or without the use of an internal bra or a sling.

Goulian et al. first described the concept of “dermal mastopexy” in 1971 [15]. In this technique, the skin of the lower quadrants is de-epithelialized, the NAC is transposed superiorly, and the dermis folds upon itself during closure of the skin envelope. However, the long-term breast suspension achieved through the dermis alone is not long lasting.

Colwell et al. published a cadaveric (AlloDerm) or autogenous dermal sling technique [16]. They used the dermal sling only on the superior pole for suspension or the circumferential sling to support the inferior pole without long-lasting numerical results. This study presents a low-cost breast surgical technique with minimal morbidity for mastopexy patients. Downward vertical dislocations were recorded in both subgroups (Table 4). Greater retardation of nipple movements was noted in the MG with a mesh sling. The median sternal notch-to-nipple distance difference from the symmetrization surgery to the last follow-up was 1 cm in the MG and 3.5 cm in the NMG. The statistically significant difference between the two groups in all measurements strengthens the idea of a long-lasting result, as already observed in clinical follow-up.

Graf et al. reported a new method for pectoralis muscle flaps used as slings and concluded that this technique provides a suitable alternative approach for long-term results in breast parenchyma suspension [14]. According to that study design, clips were placed on the parenchyma flap for objective measurement. Periodic radiological examinations were performed at one, three, six, and 12 months and then at 10 years postoperatively to analyze the breast flap and any migration with respect to the three titanium clips placed intraoperatively on the chest wall parenchyma flap. In the sling group, parenchyma migration was less than that in the control group. Similar findings were observed with the use of the ULTRAPRO^®^ sling mastopexy technique. According to our results, different grades of asymmetry appeared in both subgroups. However, glandular tissue is supported by the sling, and therefore, less variation is expected over the follow-up period. Similarly, gravity is a factor that interferes with breast ptosis. The median nipple-to-IMF distance difference from the symmetrization surgery to the last follow-up was 0.5 cm in the MG and 0.75 cm in the NMG. The mesh sling provides less tissue at the lower pole, leading to less of a bottoming-out effect, which was confirmed by this study, where the mesh sling study group maintained a longer chest flap position. Similar results were presented in the study by Graf et al. [14]. According to the radiological evaluation of breast imaging, the mesh sling did not disturb the follow-up and could be safely used in reconstructive surgery. Similar results were shown in other studies [32,33]. According to the BREAST-Q questionnaire, significantly higher scores were found in the MG for questions focused on breast satisfaction, physical wellbeing, and sexual wellbeing. Information such as the need for multiple surgeries and changes in symmetry over time should be an important part of the professional decision-making process, and the surgeon should inform the patient during surgery preparation.

Despite the currently wide popularity of oncoplastic breast surgery, due to the lack of evidence-based knowledge, the number of awaited breast surgeries, long-term symmetry, and patient satisfaction are not basic parts of the information given to patients and surgical planning. Planning for breast unit-based health systems, attention, capacity, and financial resources must also be provided to meet the long-term needs of patients undergoing PMBR. Larger series of patients are needed to confirm the long-term cosmetic results, quality of life, and patient satisfaction of this technique. The present study and standardized technical description support the ongoing prospective clinical trial with the title of Examining and Comparing the Temporal Changes and Results of Cosmetic, Quality of Life and Patient Satisfaction Achieved with Immediate and Delayed-immediate Implant-based Breast Reconstruction Procedures and Contralateral Symmetrization Techniques conducted by the National Institute of Oncology (Hungary), Department of Breast and Sarcoma Surgery, ClinicalTrials.gov, identifier: NCT04356235.

## 5. Conclusions

According to the need for long-lasting symmetry caused by the long life expectancy of early breast cancer patients, the partially absorbable ULTRAPRO^®^ mesh was used successfully to decrease the rates of pseudoptosis and nipple down-migration for breast symmetrization. This technique offers a safe and effective method to preserve breast symmetry with high patient satisfaction in the long term. The mesh sling as an internal bra seems to provide long-lasting suspension of breast tissue. Larger series of patients are needed to confirm the long-term cosmetic results, quality of life, and patient satisfaction of this technique as well as the importance of patient-related outcomes with implant-based IPMBR and contralateral symmetrization.

## Figures and Tables

**Table 1:** The patient and tumor characteristics.

Characteristics	Mesh Group	Non-Mesh Group	*p* Value
	*n* (%)	*n* (%)	
Total number of patients	59	58	
Age	49 (35–69)	48.5 (34–78)	0.735 (Mann–Whitney)
(year; median, min–max)			
Height (cm; median, min–max)	165 (154–180)	165 (135–185)	0.753 (Mann–Whitney)
Weight (kg; median, min–max)	69 (52–106)	69.5 (47–106)	0.733 (Mann–Whitney)
BMI (median, min–max)	24.6 (20.1–35)	25.7 (17.3–35.9)	0.544 (Mann–Whitney)
Smoking habits			0.749 (Fisher)
yes	7 (11.9 %)	4 (6.9 %)	
no	44 (74.6 %)	37 (63.8 %)	
no data	8 (13.5 %)	17 (29.3 %)	
Comorbidities			0.211 (chi-square)
yes	43 (72.9 %)	36 (62.1 %)	
no	16 (27.2 %)	22 (37.9 %)	
Laterality			0.307 (chi-square)
left	28 (47.5 %)	33 (56.9 %)	

BMI: Body Mass Index; ASM: Areola-Sparing Mastectomy; SSM: Skin-Sparing Mastectomy; NSM: Nipple-Sparing Mastectomy; SLNB: Sentinel Lymph Node Biopsy; ALND: Axillary Lymph Node Dissection.

**Table 2:** The surgical procedure-related characteristics.

Surgical Procedure-Related Characteristics	Mesh Group	Non-Mesh Group	*p* Value
	*n* (%)	*n* (%)	
Number of months elapsed between the primary and symmetrization surgery (month; median, min–max)	16 (3–91)	15 (5–66)	0.506 (Mann–Whitney)
Duration of operation (minutes; median, min–max)	70 (45–120)	70 (30–120)	0.371 (Mann–Whitney)
Type of surgery			0.032 (chi-square)
mastopexy	32 (54.3%)	26 (44.8%)	
breast reduction	12 (20.3%)	5 (8.6%)	
implant and mastopexy	15 (25.4%)	27 (46.6%)	
Size of the implant used on the tumour side	685 (380–1010)	620 (375–920)	0.012 (Mann–Whitney)
(cc; median, min–max)			
Shape of the implant			0.204 (Fisher)
anatomical	56 (94.9%)	51 (87.9%)	
round	3 (5.1%)	7 (12.1%)	
Size of the implant used on the symmetrization side	0 (0–225)	0 (0–325)	0.055 (Mann–Whitney)

**Table 3:** BREAST-Q postoperative results.

BREAST-Q Postoperative Questions	Mesh Group	Non-Mesh Group	*p* Value
Postop-Q1 (median, min–max)	57 (18–100)	49 (0–100)	0.0004 (Wald–Wolfowitz)
Postop-Q2 (median, min–max)	62 (34–100)	62 (37–100)	0.432 (Wald–Wolfowitz)
Postop-Q3 (median, min–max)	76 (36–100)	72 (24–100)	0.012 (Wald–Wolfowitz)
Postop-Q4 (median, min–max)	50 (14–91)	41 (0–100)	0.047 (Wald–Wolfowitz)

Postop-Q1: breast satisfaction; Postop-Q2: psychosocial wellbeing; Postop-Q3: physical wellbeing: chest; Postop-Q4: sexual wellbeing.

**Table 4:** Sternal notch-to-nipple, nipple-to-midline, and nipple-to-IMF distances.

Measurments	Mesh Group	Non-Mesh Group	*p* Value
Total number of patients	59	58	
Sternal notch-to-nipple distance on the symmetrization side at the end of the follow-up (cm; median, min–max)	22 (19.5–27.5)	23.5 (19–26.5)	0.00001 (Mann–Whitney)
Nipple-to-IMF distance on the symmetrization side at the end of the follow-up (cm; median, min–max)	7.5 (5.5–13)	6.75 (5–9.5)	0.001 (Mann–Whitney)
Nipple-to-midline distance on the symmetrization side at the end of the follow-up (cm; median, min–max)	11.5 (9–15.5)	12 (8–15)	0.118 (Mann–Whitney)
Sternal notch-to-nipple/neo-NAC distance on the mastectomy side at the end of the follow-up (cm; median, min–max)	21 (18.5–27)	21 (18–24)	0.354 (Mann–Whitney)
Nipple/neo-NAC-IMF distance on the mastectomy side at the end of the follow-up (cm; median, min–max)	7 (5–11)	7,5 (4,5–10)	0.733 (Mann–Whitney)
Nipple/neo-NAC-midline distance on the mastectomy side at the end of the follow-up (cm; median, min–max)	10.5 (9–15)	10,5 (8,5–15)	0.402 (Mann–Whitney)
Sternal notch-to-nipple distance on the symmetrization side	21 (18–26)	20 (18–21)	0.000009 (Mann–Whitney)
at the first week (cm; median, min–max)			
Nipple-to-IMF distance on the symmetrization side at the first week (cm; median, min–max)	7 (5–11)	6 (5–8)	0.0002 (Mann–Whitney)
Nipple-to-midline distance on the symmetrization side at the first week (cm; median, min–max)	11 (9–15)	11 (8–14)	0.494 (Mann–Whitney)

IMF: Inframammary Fold; NAC: Nipple-Areola Complex

## Data Availability

Data is contained within the article.
